# Retraction: Abnormal plasma levels of steroids and their ratios in patients with prurigo nodularis: a pilot study

**DOI:** 10.3389/fphys.2023.1293815

**Published:** 2023-09-19

**Authors:** Frontiers Editorial Office

The journal retracts the 2022 article cited above.

Following publication, the authors contacted the Editorial Office to request the retraction of the cited article, stating that there was a mismatch between the data from the healthy and pruritic patients’ group during data analysis. Specifically, the data of five participants of the healthy group were included in the data of the pruritic patients’ group and *vice versa*. As a result, the conclusions reported in the article are no longer supported by the data. An investigation was conducted in accordance with Frontiers’ policies that confirmed this; therefore, the article has been retracted.

The authors concur with the retraction and sincerely regret any inconvenience this may have caused to the reviewers, editors, and readers of Frontiers in Physiology.

